# Pharmacological Progress of Mitophagy Regulation

**DOI:** 10.2174/1570159X21666230314140528

**Published:** 2023-04-12

**Authors:** Sheikh Arslan Sehgal, Hao Wu, Muhammad Sajid, Summar Sohail, Muhammad Ahsan, Gulnaz Parveen, Mehreen Riaz, Muhammad Saleem Khan, Muhammad Nasir Iqbal, Abbeha Malik

**Affiliations:** 1 Department of Bioinformatics, The Islamia University of Bahawalpur, Bahawalpur, Punjab, Pakistan;; 2 Department of Bioinformatics, University of Okara, Okara, Pakistan;; 3 State Key Laboratory of Agricultural Microbiology, College of Veterinary Medicine, Huazhong Agricultural University, China;; 4 Department of Biotechnology, University of Okara, Okara, Pakistan;; 5 Department of Forestry, Kohsar University Murree, Pakistan;; 6 Institute of Environmental and Agricultural Sciences, University of Okara, Okara, Punjab, Pakistan;; 7 Department of Botany, Women University, Swabi, Pakistan;; 8 Department of Zoology, Women University, Swabi, Pakistan;; 9 Department of Zoology, University of Okara, Okara, Pakistan

**Keywords:** Mitophagy, pharmacology, mitochondria, neurological disorders, power management system, mitochondrial quality control

## Abstract

With the advancement in novel drug discovery, biologically active compounds are considered pharmacological tools to understand complex biological mechanisms and the identification of potent therapeutic agents. Mitochondria boast a central role in different integral biological processes and mitochondrial dysfunction is associated with multiple pathologies. It is, therefore, prudent to target mitochondrial quality control mechanisms by using pharmacological approaches. However, there is a scarcity of biologically active molecules, which can interact with mitochondria directly. Currently, the chemical compounds used to induce mitophagy include oligomycin and antimycin A for impaired respiration and acute dissipation of mitochondrial membrane potential by using CCCP/FCCP, the mitochondrial uncouplers. These chemical probes alter the homeostasis of the mitochondria and limit our understanding of the energy regulatory mechanisms. Efforts are underway to find molecules that can bring about selective removal of defective mitochondria without compromising normal mitochondrial respiration. In this report, we have tried to summarize and status of the recently reported modulators of mitophagy.

## 1 GENERAL INTRODUCTION OF MITOCHONDRIAL QUALITY CONTROL

Mitochondria are also known as the “powerhouse” of the cell because those organelles are key sites of the production of ATP for the survival of the cell and also for numerous significant cellular functions. Mitochondria are considered as the major executioner of cell death including necrotic and apoptotic death of cells. So, the quality and health of the mitochondria should be controlled efficiently to avoid unnecessary death of the cell. Mitochondria utilized numerous mechanisms to maintain their biogenesis and homeostasis. The injured mitochondria can generate the mitochondrial spheroids to attain the lysosomal markers activating an alternative pathway to remove the injured mitochondria [1-3]. Mitochondria constantly perform the fusion and fission processes to repair the injured mitochondrial components to exchange the stuff among healthy mitochondria through the fusion process while the separation of injured mitochondria through the fission process [4, 5].

This fission and fusion of mitochondria are quite necessary for cell survival and adaptation to changing conditions [4, 6]. The proteosome degraded the damaged proteins of outer mitochondrial membranes [7]. The proteolytic system of mitochondria helps to demolish the misfolded proteins leading to the disruption of the mitochondrial function [8, 9]. The autophagosome wrapped the injured mitochondria to activate the degradation in the lysosome through mitophagy [10-12]. Under oxidative stress, mitochondria can bud off and form mitochondrial-derived vesicles (MDV) for the degradation of oxidized proteins of mitochondria within the MDV by fusing with lysosome [13]. Notably, autophagy is linked to a number of pathophysiologic circumstances in which this cellular process either plays a cytoprotective or cytopathic role in response to a number of pressures including metabolic, inflammatory, neurodegenerative, and therapeutic stresses [14]. In response to several types of metabolic stress, such as food scarcity, growth factor depletion, and hypoxia, autophagy is induced as an adaptive catabolic mechanism [15] and used in the regulation of many *de novo* pathways. As a result, autophagy may represent a new pharmacologic target for drug development and therapeutic intervention of various human disorders [16]. It is now generally accepted that modulating the activity of autophagy through targeting specific regulatory molecules in the autophagy machinery may impact disease processes. Small molecule drugs that induce or inhibit autophagy have shown promising results in the treatment of conditions like cancer [17]. Depending on the situation, increasing either cell survival or death-two important events targeted by treatments for a variety of disorders-by inducing or suppressing autophagy may have therapeutic effects [18]. The development of autophagy-based therapeutic approaches for a number of human disorders may be facilitated by a better understanding of the biology of autophagy and the pharmacology of autophagy modulators.

## 2 RATIONALE MITOCHONDRIAL DRUG TARGETS

The rationale for mitochondrial drug targets to gain the therapeutic targets lies in the fact that mitochondria have a significant role in apoptosis, ROS production, and the regulation of energy metabolisms. Hence, the delivery of various specific drugs against mitochondria may have the ability to provide a significance treatment for numerous mitochondrial diseases to regulate and deregulate their functions. Numerous potent therapeutic applications to target mitochondrial diseases include: (a) in diabetes or obesity, activate the uncoupling proteins (UCPs), or to target the drugs to uncouple the electron transport chain (ETC), (b) in stroke and heart attack, the drug delivery against mitochondria for the inhibition of mitochondrial permeability transition (MPT) for the tissue injuries related to IR, (c) in cancer therapy, to target Bcl-2 proteins or various toxic drugs to trigger the process of apoptosis and (d) the antioxidants delivery against mitochondria for the prevention of oxidative damage linked with diabetes, IR tissue injury, and neurodegenerative diseases.

## 3 MITOCHONDRIAL FUSION AND FISSION

Mitochondria are the dynamic organelles of the cell having continuous processes of fission and fusion that lead to the survival of the cell, adaptation to changing environments for cell growth, cell division, and also mitochondrial distribution during differentiation [4]. In mammals, the fusion of the mitochondria is mediated by optic atrophy 1 (OPA1) and also through the fusion proteins mitofusin 1 (Mfn1) and mitofusin 2 (Mfn2). These proteins are dynamin-related GTPase and the fusion inner mitochondrial membrane is fused by OPA1 while Mfn1 and Mfn2 are responsible for the fusion of outer mitochondrial membranes. The paraplegin [19] (AAA protease located in the matrix of mitochondria) and presenilin-associated rhomboid-like (PARL) [20] induce alternative processing and alternative splicing of OPA1 to form 8 different variants of OPA1. Although, the processing of OPA1 still occurs in the knockout MEF cells of paraplegin or PARL suggesting the involvement of other factors in OPA1 processing [20]. Under normal conditions, OPA1 can further cleave by YME1 to form long and short forms of OPA1 (L-OPA1 and S-OPA1) [21], while the S-OPA1 is present in intermembrane space and L-OPA1 is in the inner membrane. The mitochondrial uncoupler CCCP depolarizes the mitochondria and the inducible protease OMA1 further cleaves the L-OPA1 leading to the fragmentation of mitochondria by preventing the fusion of the mitochondria [22, 23]. SIRT3, the deacetylase of mitochondria has the capability to deacetylate the OPA1 and eliminate the activity of GTPase [24]. In mammals, the fission of the mitochondria is mediated through dynamin-related protein 1 (Drp1). Drp1 is a large GTPase and a cytosolic protein that helps in mitochondrial constriction leading to dividing the mitochondrion into two mitochondria. The four different receptor proteins of mitochondria named MID51, mitochondrial dynamics protein of 49 kDa (MID4 9), mitochondria fission 1 (Fis1) and fission factor (Mff) interacted with Drp1. In mammalian cells, the interaction between Drp1 and Fis1 has not a prominent role in mitochondrial fission regulation while the Drp1 interaction with the rest of the three proteins has a significant role in fission [25-29]. Drp1 has also the ability to localize at the contact site of mitochondria to the endoplasmic reticulum, which may have a key role in the fission of the mitochondria [30]. The transcription of Drp1 is elevated by p53 which increased transcription upon binding with the promoter of Drp1 [31]. DNA-dependent protein kinase catalytic subunit (DNA-PKcs), the key regulator for the homeostasis of mitochondria, play a vitol role in the activation and phosphorylation of p53 which ultimately enhance the mitochondrial fission [31].

## 4 MITOCHONDRIAL PROTEIN QUALITY CONTROL

Numerous independent mechanisms exist to ensure the homeostasis of mitochondria from molecular to organellar level. The conserved mechanisms of Mitochondrial Quality Control (MQC) can maintain the proteome of mitochondria to promote normal functions and the survival of the cell [8, 31-36]. Due to the mitochondrial inherent vulnerability to different biochemical stresses, various MQC mechanisms have been evolved that clear or repair the damaged mitochondria. The protein MQC (PMQC) molecular level has a network of evolutionary conserved chaperons and proteases of mitochondria distributed across the compartments of the mitochondria, also the proteolytic cytosolic systems as ubiquitin-proteasome system linked with the outer membrane [7, 36-38]. PMQC has ATP-dependent chaperons of mtHSP60 and mtHSP70, responsible for folding, disaggregation, and the sorting of proteins in the compartment of the matrix of the mitochondria [39, 40]. Likely, the HSP90-type and HSP70-type chaperons activate in cytosol leading to the prevention of protein aggregation and promoting the transportation of unfolded nascent or newly synthesized polypeptides into the mitochondria [41-43]. The PMQC proteolytic facet includes numerous conserved proteases [8, 32, 33, 44].

Generally, the distribution of proteases across the sub-compartments of mitochondria can be characterized by two different groups ATP-independent proteolytic enzymes and ATP-dependent proteases (also called AAA+). Lon/Pim1 and ClpXP proteases are present in the mitochondrial matrix and degrade the aggregated or oxidatively damaged polypeptides in the mitochondrial compartment [45-52] and the inner mitochondrial membrane has two AAA+ proteases. To conduct the quality control function, the active site of matrix AAA (m-AAA) protease is exposed to the inner mitochondrial matrix side [53] while the active site of intermembrane space AAA (i-AAA) peptidase exposes to the intermembrane space [54, 55]. These are important proteases to remove the dysfunctional proteins that are linked with or intrinsic to the inner mitochondrial membrane [53-57]. Typically, the AAA+ proteases are present in the form of hetero-oligomeric (ClpXP, m-AAA) or homo-oligomeric (i-AAA, Lon/Pim1) complexes [58-61].

The Ubiquitin protease system of cytosol also represents significant features of PMQC. The Ubiquitin protease system participates in quality control and also in the removal of various mitochondrial-targeted proteins during or before their import into the organelle [32, 38, 62]. The Ubiquitin protease system can also access the sub-proteome of the outer mitochondrial membrane leading to the degradation and retro-translocation of outer mitochondrial membrane proteins and the process is known as mitochondria-associated degradation (MAD) [7, 37, 38]. This process relies on Cdc48/p97 AAA+ protein [37, 63] involved in ubiquitylated protein extraction from the endoplasmic reticulum [64, 65].

Fission and fusion mediate the dynamics of organelles followed by the facilitation of mitochondrial biogenesis and also redistribute the proteome and mtDNA throughout the network of the mitochondrial [66-69]. Due to the fusion of numerous mitochondria, such redistribution allows the replenishment of depleted components and the dilution of damaged molecules [67, 69]. The stress-induced mitochondrial hyperfusion [70] phenomenon showed the coupling between cellular stress response and mitochondrial fusion and also highlights the significance of mitochondrial dynamics. Upon the homeostatic loss as starvation or oxidative stress, highly interconnected networks built among the mitochondria in the stressed cells leads to an increase in ATP production, mitochondrial protection from autophagic removal, and content mixing [70-73].

The damaged organelles are also cleared from the network by different fragmentation events (fission) when the stress protection through the hyperfusion of the mitochondria is not enough [5]. The mitochondrial fission process helps to increase the mitochondrial number in the cell before cellular division or biogenesis of the mitochondria, and also to segregate the depolarized or dysfunctional mitochondria away from the healthy mitochondrial network [5, 69, 74]. The depolarized or damaged mitochondria segregate and the outer mitochondrial membrane sub-proteome components involved in generating the contact sites with other mitochondria are proteolyzed and ubiquitylated through the Ubiquitin proteome system to thwart their connection with healthy mitochondria [75-78]. Subsequently, the damaged mitochondria are removed through mitochondria-specific autophagy called mitophagy [79, 80].

## 5 MOLECULAR MECHANISM OF MITOPHAGY

Mitochondrial autophagy, also called mitophagy, manages the removal of unessential or dysfunctional mitochondria from cells and is considered a significant tool for both cellular and mitochondrial homeostasis [81, 82]. The interrupted mitophagy leads to the accumulation of the impaired organelles in the cell, which would consequently cause chronic diseases including liver, cancer, and cardiovascular diseases, and also neurodegenerative disorders [83].

Both Ubiquitin (Ub)-independent and Ub-dependent pathways are instrumental in mitophagy [84]. The Ub-dependent mitophagy depends on the ubiquitination of the mitochondrial surface proteins, acting as a recognition signal for the autophagic machinery of the cell. The execution and activation of the PTEN-induced putative kinase 1 (PINK1)-parkin pathway is considered a widely studied mitophagy mechanism yet. The dissolution of mitochondrial membrane potential (ΔψM) stabilizes the Ser/Thr Kinase PINK1 leading to the accumulation of Ser/Thr Kinase PINK1 on the outer mitochondrial membrane (OMM) of the mitochondria (Fig. **2D**) [85]. Parkin and its ubiquitinated substrate are both phosphorylated at Ser-65 by PINK1, activating parkin as an E3 Ub ligase [86, 87]. The parkin performs with PINK1 after getting localized on mitochondria to amplify the primary signals by bedecking the mitochondria with the Ub chains, phosphorylated by PINK1 [87]. Gp78 [88] and SMURF1 [89] have also been reported for their significant contribution to mitochondrial priming. Furthermore, NDP52 and OPTN operate as molecular adaptors, binding Ub-labeled mitochondria onto autophagosomes by direct contact with light chain 3 (LC3) *via* their LC3-interacting domains (LIR).

Optineurin (OPTN) and Nuclear Dot Protein 52 (NDP52) are recruited when the parkin Ub substrate builds up at Ser65 chains on OMM. These proteins also assist in the formation of phagophores near mitochondria by attracting the WD repeat domain, phosphoinositide interacting 1 (WIPI1), double FYVE domain-containing protein 1 (DFCP1), and unc-51-like autophagy activating kinase 1 (ULK1, autophagy-initiating factors) [87].

**Fig. (1):**
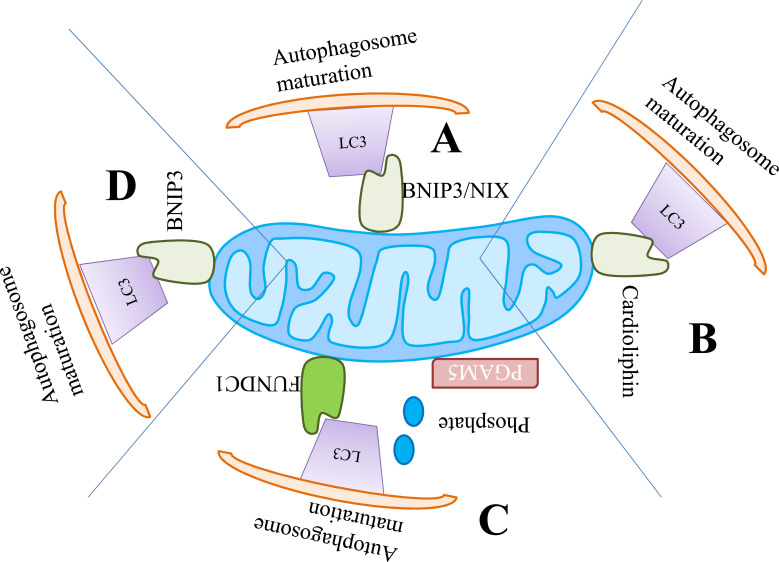
Mitophagy mechanism independent from PINK1-parkin (**A**) the process of mitophagy mediated through the accumulation of BNIP3 and NIX on mitochondria (**B**) Cardiolipin on the surface of the mitochondria triggers a mitophagic response (**C**) PGAM5 coordination with FUNDC1 leads to recruit the autophagosomes through LC3 (**D**) Mitophagy induction through BNIP3.

Additionally, the OMM localized mitophagy receptors NIP3-like protein X (NIX)/BNIP3L10 [90], BCL2/adenovirus E1B 19 kDa protein-interacting protein 3 (BNIP3) [91], and FUN14 domain-containing 1 (FUNDC1) [92], unlike mitochondrial ubiquitination, target the mitochondria to autophagosomes by direct interaction with GABAA receptor-associated protein (GABARAP) and LC3 by atypical or typical motifs of LIR. Originally, NIX was reported as a significant mediator for mitochondrial removal during the maturation of erythrocytes [93]. However, NIX has recently been implicated in ΔψM-loss-induced-mitophagy [94] (Fig. **1A**).

FUNDC1 along with BNIP3 is reported as the key mediator of hypoxia-induced mitophagy [92, 95]. Hypoxia-inducible factor 1 (HIF-1) partially controls the expression of both BNIP3 and NIX. The upregulation of NIX and BNIP3 during hypoxia helps to clear the damaged mitochondria [95, 96]. In contrast to BNIP3 and NIX, FUNDC1 expression is not dramatically affected by mitochondrial depolarization or hypoxia, but FUNDC1 function is dependent on post-translational modifications (PTM) [97]. The interactions between FUNDC1 and LC3 are considerably suppressed through the phosphorylation at various multiple sites closely located to the domain of LIR in basal conditions. Upon the collapse of ΔψM or in hypoxia conditions, FUNDC1 dephosphorylation at Ser-13 through Ser/Thr phosphatase phosphoglycerate mutase family member 5 (PGAM5) aids in the anchoring of mitochondria onto autophagosomes and enhances the binding affinity towards LC3 [98] (Fig. **1C**).

The progressive understanding of mitophagy is achieved by a small number of reported chemical approaches and tools. The number of reported inducers of mitophagy is in actual mitochondrial toxins that cause mitophagy by the way of inhibiting mitochondrial respiration. Despite off-target and significant toxicity to overall cellular biochemistry, these chemical agents are still vastly used in mechanistic and regulatory studies of mitochondrial biology and associated cellular processes. Table **1** provides comprehensive details on natural metabolites and drugs that have therapeutic effects against mitophagy-based disorders, as well as the underlying mechanism.

## 6 MITOPHAGY INDUCERS

The mitochondrial toxin, paraquat activates the process of mitophagy through excessive complex-i-dependent superoxide generation [85, 99]. The mitophagy induced by paraquat is instigated by the depolarization of mitochondria, analogous to other toxins, and operates through the PINK1-parkin pathway. The other reported toxins of parkinsonian are 6-hydroxy dopamine (6-OHDA), 1-methyl-4-phenylpyridinium (MPP+), and rotenone which stimulates mitophagy through different other ways [100-102]. In particular cases, the mitochondrial toxin rotenone causes mild depolarization of mitochondria, resulting in insufficient induction of PINK1- mediated mitophagy in neuroblastoma cells or primary neurons [101]. 6-OHDA, also staurosporine and rotenone activate the externalization process of cardiophilin that helps to recruit the autophagic machinery through its direct interactions with LC3. Generally, neurons show poor response to CCCP- and FCCP-induced degeneracy of Δψ_M_ evident through delayed parkin translocation (Fig. **1B**) [103].

Valinomycin is considered a worthy example of having highly specific ionophores of potassium (K^+^) with high lipophilicity and masking the charge of K^+^ ions through a reversible reaction by transporting them across the inner mitochondrial membrane (IMM) [104]. Salinomycin enhanced autophagy as the formation of autophagosomes, increased the number of lysosomes and LC3 flux in primary cells and cancer cell lines, and showed strong induction of autophagy as compared to rapamycin [105]. Antibiotic antimycin A is another example that elicits mitophagy response, by inhibiting respiratory complex III, increasing the Reactive Oxygen Species (ROS) level [106]. The mitophagy activated by the combination of oligomycin and antimycin A is considered a naturally activated mitophagy as both the inhibitors mediate lesser toxic effects and are quite selective to their targets leading to calculated damage to mitochondria [87, 104]. A variety of chemical substances, including paraquat, retigeric acid B, diquat, and sodium selenite, have been shown to stimulate mitophagy due to rising oxidative stress levels [85, 99, 107-109].

## 7 IONOPHORES

The proton (H^+^) ionophores, also known as protonophores, are seemingly the most widely used mitophagy inducers in cellular biological studies. The dinitrophenol (DNP), carbonyl cyanide-p-(trifluoromethoxy) phenylhydrazine (FCCP), and phenylhydrazones carbonyl cyanide m-chlorophenyl hydrazone (CCCP) have extensively been used to understand the mitophagy mechanisms and also for the characterization of involved biological pathways [87, 99, 110, 111]. CCCP was particularly considered as the first reported agent for mitophagy induction and also, was employed successfully to understand the PD-associated protein Parkin involvement in the regulatory process [99]. Even at a concentration too low to induce mitophagy, FCCP can have a significant cytotoxic effect [112]. A novel reported second-generation protonophore, named BAM15, is less toxic than FCCP but equally potent [113].

## 8 MITOPHAGY ACTIVATORS

**Fig. (2):**
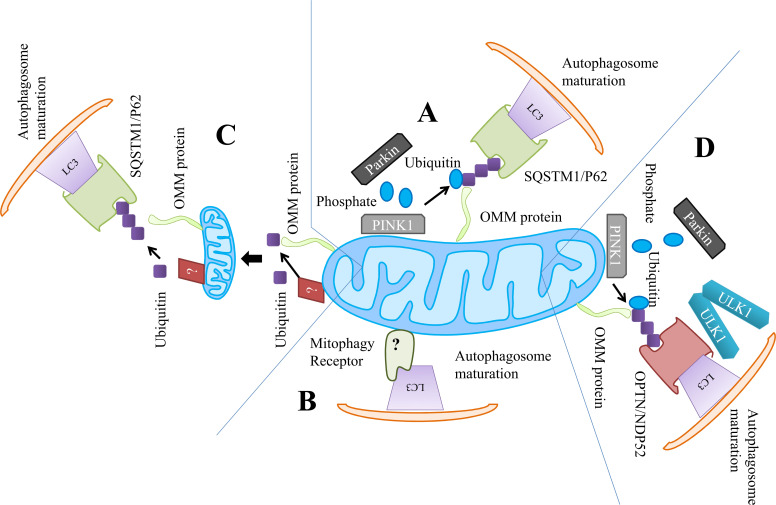
Mitophagy induction models (**A**) The mechanism of iron chelators to complete the autophagic mitochondrial removal (**B**) Unknown ubiquitin mechanism (**C**) the induction of mitophagy through PMI promotes the autophagosomal targeting of mitochondria for disposal (**D**) ubiquitinating the proteins of mitochondria to provide more ubiquitin substrate.

Nrf2 has been identified as an appealing target for improving mitochondrial health and function. Another small compound, called p62/SQSTM1-mediated mitophagy inducer (PMI), has been reported to increase the p62/SQSTM1 expression level and push mitochondria into autophagy without dissipation of Δψ_M_ (Fig. **2C**). By interfering with Nrf2's regulatory protein-protein interaction with Kelch-like ECH-associated protein 1 (Keap1), it activates Nrf2 [114]. The isothiocyanate sulforaphane and its analogues are described as the traditional inducers of Nrf2 [115]. The general autophagy induced by sulforaphane is orchestrated through a ROS-dependent mechanism, with little or no effect on Nrf2 [116].

The nicotinamide (NAM) has also been identified for mitophagy promotion without compromising Δψ_M_ or disturbing the mitochondrial function [117].

The human primary fibroblasts treatment with NAM exhibits the fragmentation of the mitochondrial network accompanied by elevated levels of resting Δψ_M_ [146]. Fisetin, Resveratrol, and the minuscule synthetic molecule SRT1720 are SIRT1 activators that have been shown to reduce the size of the mitochondrial network to a level equivalent to NAM without increasing Δψ_M_. This proves that NAM-induced hyperpolarization is independent of its effects on SIRT1 [146].

The energy restriction also activates the SIRT1 and upregulates the downstream FoxO3 effector BNIP3, thereby enhancing mitophagy in primary renal proximal tubular cells (Fig. **1D**) [147]. The mitophagy regulation through SIRT1 is activated exclusively by a receptor-based mechanism and the PINK1-parkin pathway is not required to be functionally active. Olaparib (also known as AZD2281), a PARP-1 inhibitor, has been shown to stimulate mitophagy in breast tumor cells, resulting in a reduction in the size of the mitochondrial network, which is connected to defective mitophagy (Fig. **2A**-**D**) [148, 149].

**Fig. (3):**
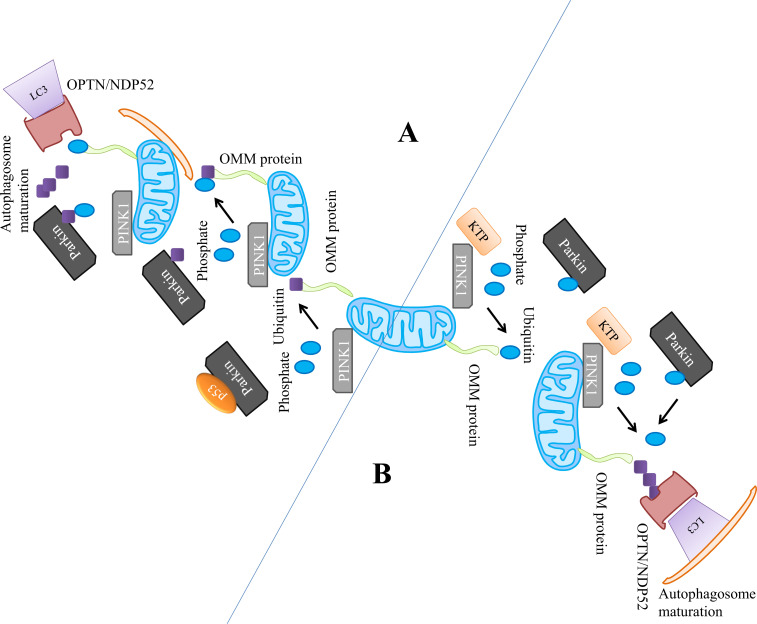
Ub-dependent mitophagy activation through PINK1–parkin modulator pathway. The mitophagy pathway of PINK1-parkin can be altered through pharmacological approaches (**A**) the utilization of pifithrin-a (small-molecule p53 inhibitor) made a pharmacological intervention to restore the clearance of mitochondria (**B**) a cell-permeable precursor, the increase of PINK1 kinase activity enhances the recruitment of parkin and mitochondrial phosphorylation.

The mitophagy may suppress by the direct interaction of parkin with p53 and halting the parkin translocation to mitochondria, though an inhibitor of p53 named pifithrin-a restores the mitochondrial clearance in a p53-dependent manner [150]. Pifithrin-a was also reported to trigger mitophagy and improve the dysfunctional mitochondria in the mouse models of both the types of diabetes such as type I and II (Figs. **3A** and **B**) [151].

PARK2 transcription modulators were identified by high throughput screening (HTS) of biologically active molecules utilizing NanoLuc and firefly coincidence reporter system [152]. These modulators also include the small inhibitors of cholesterol biosynthesis including fluvastatin and epigenetic mechanism modulators such as bromodomain and histone deacetylase [153].

A mitochondrion-located deubiquitylase named ubiquitin-specific peptidase 30 (USP30) is identified for its significant role in mitochondrial morphology regulation [154, 155]. Another molecule named 1,10ʹ-phenanthroline (Phen) having properties like siderophore was reported as a “Lead” compound in a chemical screening for the activators of mitophagy [156]. Ciclopirox olamine, an iron chelator, and Phen were identified to enhance mitophagy in response to the depolarization of the mitochondria in nematodes and various mammalian cells [157].

## 9 MITOPHAGY INHIBITORS

The widely utilized protocol for the *in vitro* blocking of the mitophagy process depends on the lysosomotropic agent's hydroxychloroquine and chloroquine or the inhibition of lysosomal acidification by employing vacuolar-type H+-ATPase (V-ATPase) inhibitor bafilomycin A1 [158]. The class III PI3K inhibitor 3-methyladeninthe, a prominent example of the compound that can inhibit autophagosome formation can suppress general autophagy and also halt mitophagy.

The mitochondrial biologists are trying to identify novel strategies and compounds to inhibit mitophagy in more specific manners but still the application has limitations. In this context, FUNDC1 LIR domain-based peptide inhibitors of receptor-driven mitophagy were discovered [98]. The phosphorylation of Ser13 residue in the LIR binding region controls FUNDC1 association to LC3 as well as its capacity to recruit mitochondria towards autophagosome [159].

Another method for inhibiting mitophagy is to prevent mitochondrial fragmentation, also linked with mitophagy [160]. In the presence of acute mitochondrial stress, substances that can block Drp1 activity, stop mitochondrial division, and stop the onset of mitophagy are also useful. The yeast cells were used for the phenotypic identification of the allosteric modulator of Drp1, named mitochondrial division inhibitors (mdivi) [161]. These inhibitors exhibited the *in vivo* cardioprotective effects by the blockage of mitochondrial fission and also preventing abnormal mitophagy to some extent [162].

The formation of autophagosomes *via* specified molecular machinery is evolutionarily conserved across different species from yeasts to higher eukaryotes. LC-3 system similar to the Atg-8 conjunction system exists in mammals. The first identified Atg-8 homologue in mammals was microtubule-associated protein light chain 3 (LC3) a light chain of microtubule-associated proteins 1A and 1B in the brain of rats [163]. The homologues of mammalian Atg-4 (mAtg-4) cleaved the C-terminal of LC3 analogous to the Atg-8 system in yeast [164]. LC3-I, the processed form of LC3 is localized in the cytosol and its C-terminus has the conserved glycine residue [164]. The homologues of mammalian Atg-3 (mAtg-3) [165] and mammalian Atg-7 (mAtg-7) [166] activate the LC3-I to form LC3-II, which is similar to the PE-conjugate [167].

Topologically, LC3-I has a central β-sheet composed of four strands (β-1, β-2, β-3 and β-4), surrounded by four α-helices (α-1 α-2, α-3, α-4). β-1 and β-4 strands are parallel to each other in the middle of the sheet and the remaining β-2 and β-3 strands are anti-parallel to β-1 and β-4 respectively [168]. LC3-I contains distinct C-terminal (30-120 residues) and N-terminal (1-29 residues) sub-domains [168], however, the whole length sequence and not either of these domains alone, interact with p62 [169]. Moreover, conformational changes in LC3-I, preceding its conjugation to PE, are similar to those seen with GATE-16 and GABARAP [170-172]. With the hydrophobic cores, a globular conformation is formed by the folding of these two sub-domains [168]. The two α-helices of the N-terminal sub-domain cover the surface of the C-terminal subdomain to form one hydrophobic core and the central β-sheet, α-3 and α-4 surrounded by the sub-domain of the C-terminal to form another hydrophobic core [168].

The rat LC3-I structure consists of two pairs of α-helices present around the five central β-sheets. The key residues (30-117 residues) contain two α-helices and five β-sheets while the remaining two helices are linked with N-terminal [173]. Till now, all the reported receptors are shown to interact with a tetrapeptide conserved motif known as LC3 Interacting Region domain (LIR) with Atg-8 proteins. The LIR domain has a core sequence of AXXB, where A is considered an aromatic residue while B is a highly hydrophobic residue and X can be any amino acid [174, 175]. The β-2 and LIR domain forms an intermolecular β-sheet stabilized by the interaction of two hydrophobic pockets and the LIR domain on the surface of the Atg-8 proteins [176].

Mitophagy is mediated by Bnip3, Nix, and FUNDC1 proteins in mammalian cells and removes either superfluous or damaged mitochondria to maintain a healthy environment in the cell [92, 177-179]. A key mitophagy receptor for mitochondrial clearance during reticulocyte terminal differentiation has been identified as the OMM protein Nix [93, 180]. Nix can push mitochondria towards autophagosomes through direct interaction with Atg-8 family proteins through the LIR domain present on its N-terminal exposed to the cytoplasm [90]. Likely Nix, its homologue Bnip3 also shows the same behaviour and behaves as a mitophagy receptor [178, 181].

Nix and Bnip3 are both found in the endoplasmic reticulum (ER) and the mitochondria, where they are involved in programmed necrosis or the regulation of apoptotic cell death by influencing ROS generation or mitochondrial respiration [182, 183]. Any mutations in the LIR domain of these proteins lead to disruption of their interactions with LC3.

FUNDC1 is a mitophagy receptor and OMM protein having three transmembrane domains with a typical LIR domain exposed to the cytosol at the N-terminal region [92]. The LIR domain's conserved residues (Y18 and L21) are critical for the connection between LC3 and FUNDC1, and deletion or mutation of these residues disrupts the LC3 interaction with FUNDC1 [92]. FUNDC1-mediated mitophagy in response to hypoxia leads to a significant reduction in the FUNDC1 at both protein and mRNA levels [92].

In mammalian cells, FUNDC1-induced mitophagy is regulated at the post-translational level by reversible phosphorylation and dephosphorylation [31, 92, 159]. In response to hypoxic stress, the FUNDC1 amino acids Ser13 and Tyr18 become dephosphorylated resulting in the loss of membrane potential [98]. Normally, CK2 phosphorylates the Ser13 and Src kinase phosphorylates the Tyr18 of FUNDC1 [98]. Collective inhibition of both these kinases is required for the activation of mitophagy [98, 159].

The FUNDC1-induced mitophagy is negatively regulated by phosphorylation though it is in contrast to other LC3 mitophagic receptor proteins which enhance the induction of mitophagy after binding with LC3 [92, 98]. Zhou [31] investigated that DNA-Pkcs also plays an important role in repressing the mitophagy by FUNDC1 by enhancing the CK2 expression due to increased phosphorylation of p53 and upregulating transcription of Drp1. In another study, Zhou, [143] revealed that FUNDC1-dependent mitophagy and platelet activation is downregulated by the therapeutic effect of melatonin against cardiac ischemia/reperfusion injury. Under the stress of FCCP or hypoxia, FUNDC1 Tyr18 and Ser13 are dephosphorylated to enhance the LC3 interaction while Ser17 becomes phosphorylated leading to the elimination of damaged mitochondria [92, 98, 184]. Thus, phosphorylation plays an important role in the regulation of mitophagy.

The LC3-II side chain (Lys49) bears structural rearrangements to bind with the FUNDC1 pSer17 peptide [185] while LC3-II also undergoes conformational changes when bound with the unphosphorylated receptors [176]. The molecular switch of LC3-II Lys49 is a classic example of phosphorylated peptide interaction [185] which has also been reported in the LC3I-Atg13 complex context [176]. The functional and structural studies reveal that the phosphorylated Tyr18 negatively regulates the interaction of FUNDC1 and LC3. Therefore, this interaction is considered a significant molecular switch for mitophagy [186].

BCL2L1 specifically suppresses receptor-mediated mitophagy, a novel regulatory hypoxia-induced mitophagy mechanism. Normally, the BCL2L1 interacts with PGAM5 for its phosphatase activity inhibition and prevents FUNDC1 dephosphorylation, leading to mitophagy activation while the stress conditions (FCCP and hypoxia) degrade the BCL2L1 resulting in PGAM5 activation and subsequent dephosphorylation of Ser13 FUNDC1 [187].

The sub-lethal concentration of mitoapocynin, mitochromanol acetate and mitoquinone can induce selective autophagy in malignant breast cancer cells [188] while the autophagy inhibition with the combination of mitoquinone is cytotoxic to MDA-MB-231 cells [189]. Malignant breast cancer cells have damaged mitochondria, the mitophagy is considered a survival mechanism [188].

Urolithin A (UA) has been reported to induce potent mitophagy in rodents, mammalian cells and *C. elegans* leading to significant improvement of multiple organismal and cellular phenotypes [190]. In mammalian cells and young worms, the UA administration for a short period decreases the contents of mitochondria though maintaining the respiratory capacity at its maximum level. The treatment of UA reduces the number of mitochondria while the remaining mitochondria can maintain the need for energy and shift the respirations from CI to CII [190]. The mitophagy induction in the heart during mice's prenatal period also shifted the respiration from CI to CII [191].

In macrophages, an increase of nitric oxide synthetase 2 generated nitric oxides after stimulation with ATP and lipopolysaccharide that increased the Sestrin 2 protein. The increased sestrin 2 enhanced mitophagy by mediating the SQSTM1 aggregation and binding to Lys-63 ubiquitinated mitochondria [192]. Specific autophagic machinery is activated by sestrin 2 for mitochondrial degradation through the maintenance of ULK1 [192].

The administration of mitoQ dramatically reversed the diabetic mouse kidney tubular damage by reducing the oxidative stress in tubular cells. The tubular cell mitophagy is accompanied by the amelioration of cell apoptosis and mitochondrial fragmentation by the regulation of mitoQ [193]. In *in vivo* and *in vitro* analyses, Nrf2 regulated PINK1 by contributing to this process. In tubular cells, mitoQ mediates mitophagy through PINK1/Nef2 and also protects against hyperglycemia-induced oxidative injury [193].

## 10 SMALL MOLECULES THAT TARGET MITOCHONDRIAL FISSION (MIDIV) AND FUSION (S30)

The mitochondrial fission is mediated by Drp1 GTPase and recruited through a variety of mitochondrial outer membrane adaptor proteins followed by the aggregation at the future fission sites along with the mitochondrion [194, 195]. Drp1 presents in the form of oligomers to generate a ring-type structure around the mitochondrion and helps to separate the mitochondrion into two separate mitochondria on contraction. The function of Drp1 can be impaired through several basic approaches including genetic knockout Drp1, treatment with cell-permeable peptides against Drp1, si/shRNA mediated depletion of Drp1, dominant-negative Drp1 expression, and the treatment of pharmacological inhibitor mdivi-1 and empagliflozin. Cassidy-Stone *et al*., and Green [196] reported a cell-permeable quinazolinone as a Drp1 inhibitor in yeast and mammalian cells [161]. Mdivi-1 was discovered by performing a primary screening of 23,100 compounds library against yeast growth anda secondary screening against the morphology of mitochondria. This inhibitor reveals 1-50μM of Ki towards the GTPase activity of yeast Drp1. Mdivi-1 does not affect the GTPase activity of the isolated GYPase domain of generic Drp suggesting that there is no direct effect of mdivi-1 on the GTPase activity through the GTPase domain [161]. The mdivi-1 prevents the Drp1 association with mitochondria and also the self-assembly of Drp1 into rings, indicating that this inhibitor likely impairs the function of Drp1 allosterically and also prevents the oligomerization of Drp1 [161]. Recently, the inhibition ability of mdivi-1 against Drp1 and also its impact on mitochondrial fission have been challenged. Bordt *et al*. [197] claimed that there is no effect of mdivi-1 treatment on the morphology of mitochondria in mammalian COS-7 and primary neuron cells and observed > 1.2 mM of Ki for Drp1 GTPase activity. The mdivi-1 can inhibit the yeast GTPase activity but it cannot inhibit the mammalian Drp1 [197, 198]. Zhou *et al*. [144] revealed that treatment of empagliflozin, a sodium-glucose cotransporter 2 (SGLT2) inhibitor, supressed mitochondrial fission by activation of adenosine monophosphate (AMP)-activated protein kinase (AMPK), suppression of Drp1^S616^ phosphorylation and increased Drp1^S637^ phosphorylation.

## 11 SMALL MOLECULES FOR MITOCHONDRIAL PROTEIN QUALITY CONTROL

Spiramine A of *Spiraea japonica* derived natural compound 15-oxospiramilactone (S3) has the potential to induce mitochondrial fusion and help in the mitochondrial network restoration [199]. The S3 high dose concentration has the ability to induce apoptosis *via* the upregulation of Bim [200] or Wnt pathway inhibition [201]. S3 does not induce apoptosis at a low concentration of 2 μM and showed a different mitochondrial fusion mechanism. OPA1 and Mfns are required for the mitochondrial fusion generated by the S3 molecule, showing that this fusion is reliant on the fusion factors already present on the mitochondrial inner and outer membranes [199]. The mitochondrial fusion induced by the S3 compound does not affect the inhibition or phosphorylation of Drp1 [199] while ethacrynic acid, 15d-PGJ2, and mdivi-1 induced the elongation of mitochondria by inhibiting the mitochondrial fission mediated through Drp1 [161, 202-205].

## CONCLUSION

To restore the ability of different cells for efficient removal of dysfunctional, injured, or damaged mitochondria are crucial for the prevention of numerous disorders and diseases. So, there is a significant need to identify novel compounds for the understanding of the mitophagy process followed by the correction of unwanted defects. Moreover, the clear understanding of the regulation of the molecular mechanism of mitophagy has still not been reflected through the development of novel compounds having potent therapeutic utilization. Both researchers and clinicians are trying to characterize the regulation of the molecular mechanism of mitophagy to selectively control the quality of the cell and to improve the pharmacological approaches.

## Figures and Tables

**Table 1:** Therapeutic effects of natural compounds and drugs against disorders due to mitophagy/autophagy.

**S. No.**	**Compounds/Drug**	**Source of Compound**	**Disorder**	**Regulation Mechanism**	**References**
1	Resveratrol	Grapes, Nuts	Ischemic Brain	AMPK‐dependent manner	[118]
			Alzheimer’s Disease	Mitochondrial biogenesis	[119-121]
			Parkinson’s Disease		
			Down Syndrome		
			Parkinson’s Disease	Regulation of mitochondrial fission/fusion dynamics by overexpression of MFN2 and OPA1	[122, 123]
			Dementia		
2	Urolithin A	Microflora	Ischemic	Activated autophagy	[124]
3	Spermidine	Rice bran, Mushrooms, Soyabeans, Broccoli	Aging	Autophagy	[125, 126]
4	Curcumin	Turmeric	Alzheimer's	Increased expression of biogenesis genes *i.e*. PGC1-α, NRF-1, NRF-2, and TFAM	[127]
5	-	-	Ischemia		[128]
6	Quercetin	Fruits and vegetables	Parkinson’s Disease	Stimulated expression of PGC-1α, TFAM, and cytochrome B	[129]
7	Dihydromyricetin	Vine tea	Cerebral hypoxia-ischemia	Enhanced PGC-1α and TFAM expression	[130]
8	Epigallocatechin-3-gallate (EGCG)	Green tea	Down‘s syndrome	Activation SIRT1/AMPK/PGC-1α signaling for improved biogenesis	[131]
9	Embelin	Berries	Parkinson’s Disease	MPTP-induced neurotoxicity by activating SIRT1	[132]
10	Xanthohumol	Hops	Huntington’s Disease	Prevention of mitochondrial dysfunction *via* up-regulation of MFN2	[133]
11	Ferulic acid	Asafoetida/Hing	Parkinson’s Disease	Reduced neuronal death by inhibition of DRP1	[134]
12	Fisetin	Strawberries		Improved activity of mitochondrial complex I	[135]
13	Naringin	Citrus fruits		Enhanced activity of mitochondrial ETC complexes	[136]
14	Cannabidiol	*Cannabis sativa*	OGD/R damage	Improved mitochondrial respiration and ATP production	[137]
15	Progesterone, estradiol	Animal/human origin	-		[138]
16	α-Lipoic acid		Huntington’s Disease	Enhanced activity of mitochondrial ETC complexes	[139]
17	Niclosamide	Oral Antihelminthic Drug	Parkinson’s Disease	Enhance PINK1 stabilization to reverse Mitochondri depolarisation al	[140, 141]
18	Gemcitabine	Chemotherapeutic Drug		PINK1-dependent mitophagy	[142]
19	Melatonin	Drug	Cardiac ischemia	Platelet activation by blocked FUNDC1-required mitophagy	[143]
20	Empagliflozin	Drug	Type 2 diabetes	AMPK-mediated inhibition of mitochondrial fission	[144]
21	Metformin	Drug		Inhibition of complex I in the mitochondrial respiratory chain	[145]

## LIST OF ABBREVIATIONS

ETC: Electron Transport Chain
HTS: High Throughput Screening
MAD: Mitochondria-associated Degradation
MDV: Mitochondrial-derived Vesicles
MPT: Mitochondrial Permeability Transition
MQC: Mitochondrial Quality Control
PARL: Presenilin-associated Rhomboid-like
PTM: Post-translational Modifications
ROS: Reactive Oxygen Species
